# Decadal transition of adult mortality pattern at Ballabgarh HDSS: evidence from verbal autopsy data

**DOI:** 10.1186/s12889-015-2119-1

**Published:** 2015-08-14

**Authors:** Sanjay Kumar Rai, Arti Gupta, Rahul Srivastava, Mohan Bairwa, Puneet Misra, Shashi Kant, Chandrakant S. Pandav

**Affiliations:** Centre for Community Medicine, All India Institute of Medical Sciences, Centre for Community Medicine, 110029 New Delhi, India; INDEPTH Network, Accra, Ghana

## Abstract

**Background:**

Mortality levels and patterns are significant indicators of population health, and are of importance to prioritize the goals of health systems and efficient resource allocation. We ascertained the decadal transition of mortality pattern in adult population aged 15 years and above during the years 2002–2011.

**Methods:**

All adult deaths aged 15 years and above during the years 2002 to 2011 were included in the study. Cause of death was ascertained by verbal autopsy tool for adults which is a validated questionnaire developed at Ballabgarh Health and Demographic Surveillance System (HDSS). Cause and age specific mortality, and mean age at death was determined for individual years.

**Results:**

A total of 4,276 deaths (≥15 years) occurred in the Ballabgarh HDSS during the years 2002 to 2011. Of these, 96.8 % deaths were investigated using verbal autopsy tool. Of total deaths investigated, 60.6 % were males. Cardiovascular diseases (19.6 %) were the leading cause of death, followed by respiratory diseases (16.5 %). In the age group of 15–59 years, the most common cause of mortality was external causes of mortality (28.9 %). Most common cause of death was senility (20.8 %) in females, whereas cardiovascular diseases were commonest cause (19.6 %) in males. Road traffic injuries contributed 6.7 % deaths in males compared to 1.5 % in females. Over the years, the proportions of mortality due to cardiovascular diseases had increased (12.6 % to 18.8 %). Mortality proportions had decreased for infectious diseases (12.1 % to 9.5 %) and respiratory diseases (24.7 % to 10.9 %). Mortality due to neoplasms remained nearly stagnant (6.6 % to 6.4 %).

Mean age at death due to cardiovascular diseases and neoplasm had increased from 57 years (95 % CI: 52.2–62.9) to 62 years (95 % CI: 59.2–65.4) and 58 years (95 % CI: 53.1–63.2) to 62 years (95 % CI: 57.0–66.7), respectively, during the decade. Mean age at death had decreased for road traffic injuries and infectious diseases from 41 years (95 % CI: 31.7–50.8) to 39 years (95 % CI: 34–43.4) and 53 years (95 % CI: 48.3–58.6) to 50 years (95 % CI: 44.1–55.8), respectively over the years.

**Conclusion:**

Mortality surveillance using verbal autopsy tool revealed a transition in cause specific deaths from respiratory diseases to cardiovascular diseases over the decade. The apparent epidemiological transition in the community demands reorientation of healthcare priorities.

## Background

Developing countries generally lack consistent, timely, and reliable information on levels of cause specific mortality fractions in their populations [1, 2]. Verbal autopsy (VA) is a useful tool in such settings to establish the probable cause of death (COD) by interviewing a caregiver or a close person who provide witness to the death event [3]. Mortality levels are significant indicators of population health and are of extreme importance to prioritize the goals of health systems and efficient resources allocation [4, 5]. Most of the nine million annual deaths in India, as in most low—and middle-income countries, occur at home, without medical attention or certification [6, 7]. Validity of the VA tool has been shown to be satisfactory to fill this huge gap of information on cause of deaths in developing countries.

Over the past six decades, life expectancy at birth in India has nearly more than doubled [8, 9]. Along with increasing life expectancy and aging of population [10], mortality patterns have also changed. Increasing trends in non-communicable diseases has been observed in recent decades which are further aggravating the survival disadvantages [11]. Therefore, we ascertained the decadal transition of mortality pattern in adult population aged 15 years and above during the years 2002–2011 at Ballabgarh Health and Demographic Surveillance System (HDSS).

## Methodology

### Study setting

The Ballabgarh HDSS, also known as the Comprehensive Rural Health Services Project (CRHSP), Ballabgarh, is set up of 28 villages in Ballabgarh block of Faridabad district of Haryana in north India. Ballbgarh HDSS was established in 1961 to develop a model for rural health-care practice in India. In addition to demographic surveillance and community based research, CRHSP, Ballabgarh provides preventive, health promotion, and curative services to its surrounding population through a network of one secondary-level hospital at Ballabgarh, two primary health centres (PHC) and 12 subcentres. Ballabgarh HDSS had a population of 90,240 as on 31^st^ December 2011. More details of Ballabgarh HDSS is available in Journal of IEA published in June 2013 [12].

### Data collection methods

**Fig. 1:**
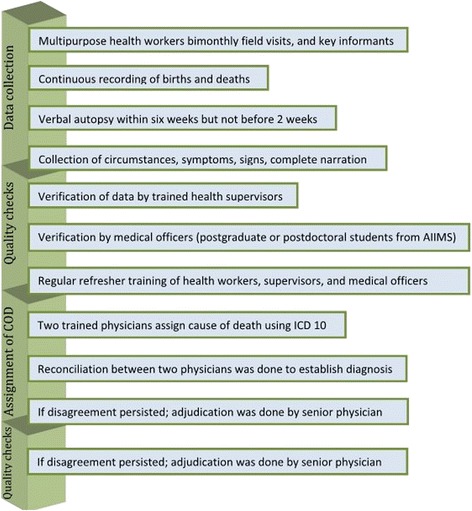
Flow chart of data collection and cause of death assignment

Multipurpose health workers (MPHWs) visit all households under the HDSS twice monthly and provide basic health services such as immunization, family planning etc. to the population under surveillance. MPHWs collect demographic and health information during the visits, and administer VA tool to all deaths for determination of the COD as a part of routine services. In addition to bi-monthly visits, MPHWs also gather information through a network of key informants and opinion leaders including the village headman (Sarpanch), childcare workers (anganwadi workers), community volunteers (Accredited Social Health Activist; ASHA), school teachers, members of women’s committees (village heath and sanitation committee, and sakshar mahila samooh), priests and cremation staff. Besides this, annual census is conducted to collect data on demographic characteristics of population in the month of December each year. MPHWs were provided training in filling up of the VA forms before introducing the VA tool in Ballabgarh HDSS and refresher training are given regularly during the monthly meetings by the Primary Health Centre medical officers (Fig. 1).

In HDSS, Ballabgarh, computerized Health Management Information System is in place since 1988 [13]. Database includes information on “residents” i.e. those who are living in the area for the last six months. Visiting relatives/out-migrants are excluded from information collection. Status of in-migrant/ out-migrant is updated annually. In the present study, all adult deaths aged 15 years and above that occurred during 2002 to 2011 were included in the study. Trained health workers administered the VA tool to all deaths occurring in Ballabgarh HDSS to ascertain probable cause of death. This VA tool was developed based on the WHO VA tool. This tool was validated in Ballabgarh HDSS in 2002 and had a good agreement with the WHO VA tool [14]. This tool is short and requires less time to administer than the WHO VA tool. This tool requires minimal training. The VA tool contained structured questions including basic socio-demographic details such as age, sex, social status (caste), date of death, place of death, time of death, care seeking before death, and symptom checklist. A verbatim narrative report included symptoms/signs and circumstances of death and medical care/treatment received before death was recorded. Quality checks i.e. double data entry, inter-village and month-wise comparison of indicators, monitoring by trained supervisors, and verification by trained physicians were applied to ensure correctness of data collection. Considering the sensitivity of the information and emotional trauma following the death of a beloved, a reasonable time period was given for grieving before administering the VA tool. This was done usually after two weeks of death but within six weeks. The tool was administered in local language (Hindi). Interviewees were close associates of the deceased, for example, son, daughter, spouse, parents, siblings, father/mother/brother/sister-in-laws, who were familiar with the events before the death.

### Assignment of cause of death

Underlying causes of death were assigned by trained physician coders using a three digit code as per the International Classification of Disease 10th Revision (ICD 10) [15]. Physician coders were trained in VA coding as per ICD-10. ICD-10 algorithms were used by them [16]. Each VA form was independently assessed by two physician coders and assigned an underlying COD according to ICD 10. In case of any disagreement between the two physician coders, a process of reconciliation was attempted. If disagreement still persisted, adjudication was done by a third senior physician. Deaths for which, a specific underlying cause was not ascertainable, were assigned the code R99.

### Data analysis

This is a secondary data analysis of deaths that occurred over the years 2002–2011. Cause and age specific mortality, and mean age at mortality was determined for individual years. Cause specific mortality (proportional) according to ICD 10 classification were calculated by age groups (15–59 years, and 60 years or above), and sex. Proportional cause specific mortality by years were determined for major categories including infectious diseases, neoplasms, cardiovascular diseases, respiratory diseases, digestive diseases, external causes of mortality, nervous system diseases, genitourinary diseases, endocrine diseases, and not classifiable. Mean age at deaths by years were calculated for major categories including infectious diseases, cardiovascular diseases, respiratory diseases, external causes of mortality, and neoplasms. Mean age at deaths for the years were estimated for six major diseases including chronic obstructive pulmonary disease (COPD), ischemic heart disease (IHD), tuberculosis, cerebrovascular accident (CVA), harms and road traffic injuries (RTI). The 95 % Confidence Intervals (CI) were calculated for the proportions and mean age at death. Data were analyzed using STATA 12 (StataCorp LP, College Station, Texas, USA).

### Ethical clearance

All information collected in the present study was a part of routine services existing in the HDSS, Ballabgarh. An informed verbal consent was obtained from the caregivers of deceased individuals. The information gathered from interviewees was kept confidential. One of co-author (SK) is the owner and custodian of database. This was a secondary data analysis, hence, ethical approval was obtained after completion of the study from the Institute Ethics Committee of AIIMS, New Delhi.

## Results

Over a period of 10 years (2002–2011), 5,777 deaths occurred among all age groups in the Ballabgarh HDSS. Of these, 4,276 deaths (74 %) belonged to age group 15 years and above; 61 % males and 39 % females. Verbal autopsy forms had been filled up for 96.8 % deaths (4,140/4,276) that occurred in the HDSS. Over the decade, age and sex composition of population had changed in HDSS Ballabgarh. Population of HDSS Ballabgarh increased from 79,830 in 2002 to 90,240 in 2011. Crude birth rate, and crude death rate went down from 29.1 to 23.2 live births per 1000 population and 6.8 to 6.7 deaths per 1000 population, respectively. In HDSS Ballabgarh, sex ratio changed from 117 to 113 males per 100 females over decade while sex ratio at birth rose from 117 to 123 males per 100 females. Over the decade, crude outmigration rate, and crude immigration rate changed from 18.1 % to 16.5 %, and 6.8 % to 7.7 %, respectively.

Of the 4140 deaths, 39 % were females and 61 % men. Of them, 58 % were aged 60 years and above (Table 1). Over the decade, major categories in which deaths occurred were cardiovascular diseases (19.6 %), respiratory diseases (16.5 %), and senility (14.7 %). Other leading categories were external causes of mortality (14.4 %), infectious & parasitic diseases (12.4 %), and neoplasms (7.2 %). Road traffic injuries contributed 32 % deaths caused by external causes of mortality. Most common cause of mortality in the age group of 60 years and above was respiratory diseases (23 %), and in the age group of 15 to 59 years was external cause of mortality (29 %). Most common cause of death in males was cardiovascular diseases (19.6 %) while senility (20.8 %) in females.

**Table 1:** Cause specific mortality (proportional) by adult age groups and sex in Ballabgarh HDSS, 2002–2011*

Causes of death as per ICD 10	15-59 years			60 years and above			Total		
	Male (*n* = 1168)	Female (*n* = 568)	Total (*N* = 1736)	Male (*n* = 1340)	Female (*n* = 1064)	Total (*N* = 2404)	Male (*n* = 2508)	Female (*n* = 1632)	Total (*N* = 4140)
Infectious & parasitic diseases (A00-B99)	205(17.6)	100(17.6)	305(17.6)	120(9)	87(8.2)	207(8.6)	325(13)	187(11.5)	512(12.4)
Tuberculosis	156(13.4)	54(9.5)	210(12.1)	68(5.1)	23(2.2)	91(3.8)	224(8.9)	77(4.7)	301(7.3)
Diarrhea	20(1.7)	16(2.8)	36(2.1)	39(2.9)	47(4.4)	86(3.6)	59(2.4)	63(3.9)	122(2.9)
Neoplasm (C00-D48)	93(8)	68(12)	161(9.3)	80(6)	59(5.5)	139(5.8)	173(6.9)	127(7.8)	300(7.2)
Cardio Vascular (I00- I99)	214(18.3)	105(18.5)	319(18.4)	277(20.7)	216(20.3)	493(20.5)	491(19.6)	321(19.7)	812(19.6)
Ischemic heart disease	143(12.2)	34(6)	177(10.2)	146(10.9)	103(9.7)	249(10.4)	289(11.5)	137(8.4)	426(10.3)
Cerebrovascular accident	22(1.9)	17(3)	39(2.2)	37(2.8)	46(4.3)	83(3.5)	59(2.4)	63(3.9)	122(2.9)
Hypertension	4(0.3)	17(3)	21(1.2)	18(1.3)	14(1.3)	32(1.3)	22(0.9)	31(1.9)	53(1.3)
Respiratory system (J00-J99)	84(7.2)	46(8.1)	130(7.5)	362(27)	192(18)	554(23)	446(17.8)	238(14.6)	684(16.5)
COPD	46(3.9)	27(4.8)	73(4.2)	251(18.7)	127(11.9)	378(15.7)	297(11.8)	154(9.4)	451(10.9)
Digestive system (K00-K93)	74(6.3)	27(4.8)	101(5.8)	40(3)	22(2.1)	62(2.6)	114(4.5)	49(3)	163(3.9)
External cause of mortality (S00-Y98)	370(31.7)	131(23.1)	501(28.9)	58(4.3)	36(3.4)	94(3.9)	428(17.1)	167(10.2)	595(14.4)
Intentional harm	105(9)	72(12.7)	177(10.2)	9(0.7)	2(0.2)	11(0.5)	114(4.5)	74(4.5)	188(4.5)
Road traffic injuries	147(12.6)	13(2.3)	160(9.2)	20(1.5)	12(1.1)	32(1.3)	167(6.7)	25(1.5)	192(4.6)
Falls	7(0.6)	2(0.4)	9(0.5)	6(0.4)	10(0.9)	16(0.7)	13(0.5)	12(0.7)	25(0.6)
Assault	7(0.6)	2(0.4)	9(0.5)	3(0.2)	0(0)	3(0.1)	10(0.4)	2(0.1)	12(0.3)
Nervous system (G00-G99)	21(1.8)	11(1.9)	32(1.8)	10(0.7)	9(0.8)	19(0.8)	31(1.2)	20(1.2)	51(1.2)
Genitourinary system (N00-N99)	15(1.3)	7(1.2)	22(1.3)	25(1.9)	9(0.8)	34(1.3)	40(1.6)	16(1)	56(1.3)
Endocrine, nutritional, metabolic (E00-E90)	11(0.9)	12(2.1)	23(1.3)	20(1.5)	12(1.1)	32(1.3)	31(1.2)	24(1.5)	55(1.3)
Unclassifiable (R00-R99 except R54)	81 (6.9)	61 (10.7)	142 (8.2)	78 (5.8)	82 (7.7)	160 (6.7)	159 (6.3)	143 (8.8)	302 (7.3)
Senility (R54)	-	-	-	270(20.1)	340(32)	610(25.4)	270(10.8)	340(20.8)	610(14.7)

*In parentheses, the given number is percentage

Yearly trends of cause specific mortality in age group of 15 years and above is shown in Table 2. Over the years, there was increase in the proportions of mortality for cardiovascular diseases (12.6 % to 18.8 %). While mortality proportions had decreased among infectious diseases (12.1 % to 9.5 %) and respiratory diseases (24.7 % to 10.9 %). Steepest decline was seen among respiratory diseases from 24.7 % (95 % CI: 20.1–29.5) to 10.9 % (95 % CI: 8.3–14.1) over the decade. Cause specific mortality had remained stagnant for digestive diseases, endocrine diseases, and neoplasms.

**Table 2:** Trend of cause specific mortality over the years (2002–11) in Ballabgarh HDSS*

Year	Infectious	Neoplasms	Cardiovascular	Respiratory	Digestive	External causes	Nervous	Genitourinary	Endocrine	Not classifiable
2002	12.1 (9.1–19.9)	6.6 (4.4–9.7)	12.6 (9.6–16.6)	24.7 (20.1–29.5)	4.6 (2.8–7.3)	13.5 (10.3–17.5)	1.4 (.6–3.3)	0.9 (0.3–2.5)	2 (0.9–4.1)	1.4 (0.6–3.3)
2003	12.7 (9.8–16.4)	5.6 (3.7–8.3)	17.3 (13.9–21.4)	20.4 (16.7–24.6)	5.1 (3.3–7.7)	13.7 (10.7–17.5)	1.8 (0.8–3.6)	2 (1.1–3.9)	1 (0.4–2.6)	2.8 (1.6–4.9)
2004	18.3 (14.7–22.7)	4.4 (2.6–7.1)	16.4 (12.9–20.6)	18.3 (14.7–22.7)	3.9 (2.3–6.4)	12.5 (9.5–16.3)	0.6 (0.2–2)	1.4 (0.6–3.2)	0.6 (0.3–2.4)	2.2 (1.2–4.3)
2005	15.9 (12.4–20)	6.1 (4–9.1)	16.4 (12.9–20.7)	19.9 (16–24.4)	4.6 (2.9–7.4)	12.4 (9.3–16.3)	1.2 (0.5–2.9)	1.4 (0.6–3.3)	0.9 (0.2–2.5)	3.2 (1.8–5.6)
2006	10.9 (8.2–14.4)	8 (5.7–11.2)	22.8 (18.9–27.2)	19.4 (15.8–23.7)	2.1 (1.1–4)	15.8 (12.5–19.8)	1 (0.4–2.6)	1 (0.4–2.6)	2.1 (1.1–4)	3.1 (1.8–5.4)
2007	12 (9–15.8)	9.2 (6.6–12.7)	36.6 (31.8–41.7)	14.2 (11–18.3)	4.2 (2.6–6.8)	11.5 (8.6–15.2)	2 (1–3.9)	0.3 (0.05–1.6)	2.2 (1.1–4.4)	1.7 (0.7–3.6)
2008	9.2 (7.1–11.9)	7.9 (5.9–10.5)	14.4 (11.7–17.6)	13.8 (11.2–17)	3.5 (2.2–5.4)	17.2 (14.2–20.6)	1.3 (0.6–2.6)	1.5 (0.8–2.9)	0.7 (0.3–1.9)	3.5 (2.3–5.4)
2009	14.6 (11.7–18)	9.2 (6.9–12.1)	22.3 (18.8–26.2)	15.2 (12.3–18.7)	2.5 (1.4–4.3)	15.4 (12.5–18.9)	0.8 (0.3–2.1)	1.3 (0.5–2.7)	0.4 (0.1–1.5)	4.2 (2.7–6.4)
2010	10.7 (8.3–13.8)	8 (5.9–10.8)	20 (16.7–23.8)	12.6 (14.9–21.8)	4.2 (2.1–5.4)	15.8 (12.8–19.3)	0.8 (0.3–2.1)	1.7 (0.8–3.2)	1.1 (0.5–2.4)	0.6 (0.2–1.8)
2011	9.5 (7.2–12.6)	6.4 (4.5–9.1)	18.8 (15.5–22.7)	10.9 (8.3–14.1)	5.1 (3.4–7.5)	13.7 (10.9–17.2)	1.6 (0.7–3.2)	1.8 (0.9–3.5)	2.7 (1.5–4.6)	0.4 (0.1–1.6)

*The numbers in parentheses are lower and upper bound of 95 % Confidence Interval

Over the years (2002–2011), increase in mean age at mortality due to cardiovascular diseases, and neoplasms was observed from 57.6 years (95 % CI: 52.2–62.9) to 62.3 years (95 % CI: 59.2–65.4), and 58 years (95 % CI: 53.1–63.2) to 62 years (95 % CI: 57.0–66.7), respectively. Mean age at mortality due to infectious diseases had declined from 53.5 years (95 % CI: 48.3–58.6) to 50 years (95 % CI: 44.1–55.8). While it had remained stagnant for deaths due to respiratory diseases, and external causes of mortality, and neoplasms (Table 3).

**Table 3:** Trend of mean age of cause specific mortality over a decade (2002–2011) in Ballabgarh, HDSS (*n* = 4140)*

Year	Infectious diseases	Cardiovascular diseases	Respiratory diseases	External causes	Neoplasms
2002	53.5 (48.3–58.6)	57.6 (52.2–62.9)	67.3 (64.3–70.3)	41.1 (35.3–46.9)	58.2 (53.1–63.2)
2003	54.5 (48.6–60.5)	58.8 (54.5–63.2)	70.8 (67.5–73.9)	36.5 (31.7–41.3)	57.3 (49.2–65.3)
2004	58.7 (54.2–63.2)	60.5 (55.5–65.4)	69.0 (66.4–71.6)	41.1 (35.2–46.9)	59.3 (48.9–70.2)
2005	56.6 (52.2–61.0)	61.7 (56.5–66.9)	66.3 (62.7–69.9)	34.4 (29.9–38.9)	51.4 (43.9–58.9)
2006	51.8 (45.2–58.5)	61.9 (57.8–65.8)	68.7 (65.9–71.6)	42.8 (37.9–47.6)	51.6 (46.2–56.9)
2007	53.0 (47.3–58.7)	65.3 (62.5–68.0)	69.2 (66.1–72.3)	41.6 (35–48.2)	58.4 (52.6–64.1)
2008	51.5 (46.1–56.9)	59.9 (55.9–63.9)	68.0 (64.7–71.2)	36.5 (32.9–39.9)	55.1 (50.9–59.3)
2009	51.8 (46.6–56.9)	60.0 (56.7–63.3)	69.8 (67.6–72.0)	38.9 (34.9–43.0)	57.3 (52.4–62.3)
2010	54.9 (49.3–60.4)	62.6 (59.6–65.7)	68.2 (65.3–70.9)	39.2 (34.9–43.4)	61.7 (57.3–66)
2011	50.0 (44.1–55.8)	62.3 (59.2–65.4)	67.4 (64.1–70.7)	38.7 (34–43.4)	61.9 (57–66.7)

*In parentheses, the two numbers are lower and upper bound of 95 % Confidence Interval

Mean age at mortality was estimated for six major diseases for the decade (2002–2011). Upward shift was observed in the mean age at mortality due to road traffic injuries, and tuberculosis in the decade from 41 years (95 % CI: 31.7–50.8) to 45 years (95 % CI: 30.6–59.5) and, 50 years (95 % CI: 44.3–55.6) to 53.3 years (95 % CI: 48.3–58.4), respectively. Mean age at mortality due to cerebrovascular accidents also increased marginally from 62.8 years (95 % CI: 50.1–75.6) to 64.5 years (95 % CI: 58.7–70.4) (Table 4).

**Table 4:** Trend of mean age of cause specific mortality of six major diseases in Ballabgarh HDSS (*n* = 4140)*

	COPD	IHD	TB	CVA	Harms	RTI
2002	67.5 (64.4–70.5)	54.3 (47.5–61)	49.9 (44.3–55.6)	62.8 (50.1–75.6)	32.1 (26.2–37.9)	41.2 (31.7–50.8)
2003	71.5 (68.5–74.5)	61.1 (56.1–66.1)	49.9 (42.1–57.8)	61.7 (53.9–69.4)	31.8 (25.3–38.3)	30.8 (32.9–46.7)
2004	68.9 (66.5–71.3)	64.1 (58.1–69.9)	50.4 (44.3–56.5)	65.1 (58.3–71.8)	28.9 (23.2–34.8)	44.6 (33.5–55.7)
2005	66.5 (63.1–70.1)	65.3 (59.8–70.8)	52.8 (48.2–57.6)	63.7 (54.8–72.5)	27.6 (22.9–32.2)	31.9 (23.1–40.7)
2006	69.9 (67.3–72.7)	64.0 (59.6–68.5)	47.7 (40.7–54.7)	64.9 (56.3–73.5)	33.7 (23.7–43.6)	44.9 (37.1–52.8)
2007	68.1 (64.5–71.5)	65.8 (62.6–69)	49.9 (42.4–57.4)	61.7 (54.3–69.1)	35.1 (24.1–46.2)	50.5 (38.7–62.3)
2008	69.1 (65.9–72.2)	58.0 (52.9–63.1)	53.3 (47.6–59.1)	66.3 (59.3–73.2)	29.8 (25.5–34.1)	36.5 (30.9–41.9)
2009	69.8 (66.6–73)	61.2 (56.1–66.4)	48.9 (43.4–54.5)	63.0 (58.1–67.9)	28 (23.5–32.5)	37.8 (32.2–43.4)
2010	67.4 (64.2–70.7)	61.0 (56.2–65.8)	54.8 (48.1–61.6)	66.6 (61.9–71.4)	33.5 (22.4–44.7)	43.8 (36.5–51.0)
2011	67.4 (64.1–70.7)	59.8 (55.5–64.2)	53.3 (48.3–58.4)	64.5 (58.7–70.4)	31.8 (25.8–37.7)	45.1 (30.6–59.5)

*In parentheses, the two numbers are lower and upper bound of 95 % Confidence Interval

## Discussion

Million death study and Global burden of disease estimates documented health transition in India [11, 17–19]. This study from north India substantiates decadal transition among adult population of Ballabgarh HDSS. Decline in crude birth and death rates was observed over the decade in the HDSS.

The males contributed 61 % of all adult deaths recorded. Similar to our study findings, a study from north India also documented that 60 % deaths were contributed by males [20]. There was a minimal change in sex ratio from 117 to 113 males per 100 females in the HDSS. This may be attributed to high sex ratio at birth and/or high female child mortality in under 5 years [21]. In addition, there was slight preponderance of males (54 %) in the population composition throughout the decade in the HDSS. Deaths due to road traffic injuries were more common in males (6.7 %) compared to females (1.5 %).

We found that cardio-vascular diseases, along with respiratory diseases and external causes, were the leading causes of deaths. Similar findings have been reported in study from urban slums of Kolkata where the leading cause of deaths were cardiovascular mortality, followed by malignancies, and respiratory ailments [4]. Mortality data from the Andhra Pradesh Rural Health Initiative documents non communicable and chronic diseases as top leading causes of deaths in rural area [22]. The cause of death study in the sample registration system (SRS) during 2001–2003 has reported about 40 % of the deaths due to NCDs in rural areas of India. The SRS cause of death study also documented that cardiovascular diseases and respiratory diseases were leading causes of deaths [23]. However, these studies did not provide time trends for causes of deaths.

We report a decline in mortality due to infectious and respiratory diseases and significant rise for deaths due to cardiovascular diseases. It is also evident from Global Burden of Disease 2010 estimates that there is shift away from communicable diseases towards non-communicable diseases in adults in India [11]. Other rural areas of India may also be witnessing similar epidemiological transition. A study from north India documented rising trend of cardiovascular diseases in rural populations [20].

Despite a remarkable decline in deaths during the decade, respiratory diseases still continued to be one of the leading causes of mortality among the persons aged 15 years and above in the HDSS. Cardiovascular diseases had emerged as the most prominent cause of death in the decade (2002–2011). Hence, specific health initiatives such as opportunistic screening for early detection of cardiovascular diseases are warranted.

Mortality burden due to neoplasms has been observed as an important cause of death in Ballabgarh HDSS. Neoplasms contributed 6.9 % deaths in males and 7.8 % in females over the study period in HDSS Ballabgarh. A nationally representative study (2001–2003) reported 8 % deaths in males and 12.7 % in females due to neoplasms [19].

Deaths due to road traffic injuries contributed around 4.6 % of total deaths in the HDSS Ballabgarh. Of these, 83 % occurred in the age group 15–59 years with 92 % deaths among male population. However, external causes of mortality did not show clearly visible change in pattern in HDSS Ballabgarh. A Million Death Study publication on road traffic injuries stated that 65 % of the total deaths occurred in males between the ages 15 and 59 years. Improved pre-hospital transport and hospital trauma care might address over a third of the deaths due to road traffic injuries. Specific interventions are needed to prevent collisions and reduce injuries that might address over half of all deaths due to road traffic injuries [18].

Mean age at death due to cardiovascular diseases and neoplasms had increased while it remained nearly stagnant for respiratory and infectious diseases, this finding is consistent with increasing life expectancy in the decade [9, 12]. Decrease in mean age at death (41.1 to 38.7 years) due to external causes of mortality during the decade needs priority attention as major fraction of deaths in this group belonged to productive age group, 15–59 years age (28.9 %) compared to 60 years and above (3.9 %). However, change in mean age at death for individual diseases provides inconclusive findings but tuberculosis. Improvement in mean age at death for tuberculosis can be attributed to the good performance of the Revised National Tuberculosis Control Programme in the country [24].

## Conclusion

Mortality surveillance incorporating verbal autopsy tool revealed a transition in cause of deaths from respiratory diseases to cardiovascular diseases over the decade. Focus of the policy makers ought to shift towards mortality due to cardiovascular diseases. The rural populations are undergoing epidemiological transition that demands reorientation of healthcare priorities.
